# Synthesis of SPIO Nanoparticles and the Subsequent Applications in Stem Cell Labeling for Parkinson’s Disease

**DOI:** 10.1186/s11671-021-03540-z

**Published:** 2021-06-14

**Authors:** Li An, Qing Tao, Yue Wu, Nana Wang, Yan Liu, Feifei Wang, Lixing Zhang, Aihua Shi, Xiumin Zhou, Shuang Yu, Jingzhong Zhang

**Affiliations:** 1 Suzhou Institute of Biomedical Engineering and Technology, Chinese Academy of Sciences, No. 88 Keling Road, Suzhou New District, Suzhou, 215163 China; 2 Zhengzhou Institute of Engineering and Technology Affiliated with SIBET, Zhengzhou, 450001 China; 3 Department of Radiology, The First Affiliated Hospital of Soochow University, Suzhou, China; 4 Department of Oncology, The First Affiliated Hospital of Soochow University, Suzhou, China; 5 Tianjin Guokeyigong Science and Technology Development Company Limited, Tianjin, 300399 China

**Keywords:** SPIO, Stem cells, Parkinson’s disease, Labeling

## Abstract

Parkinson's disease (PD) is characterized by the progressive loss of dopaminergic neurons in the midbrain, and the stem cell transplantation method provides a promising strategy for the treatment. In these studies, tracking the biological behaviors of the transplanted cells in vivo is essential for a basic understanding of stem cell function and evaluation of clinical effectiveness. In the present study, we developed a novel ultrasmall superparamagnetic iron oxide nanoparticles coating with the polyacrylic acid (PAA) and methoxypolyethylene glycol amine (PEG) by thermal decomposition method and a two-step modification. The USPIO-PAA/PEG NPs have a uniform diameter of 10.07 ± 0.55 nm and proper absorption peak of the corresponding ligands, as showed by TEM and FTIR. MTT showed that the survival of cells incubated with USPIO-PAA/PEG NPs remained above 96%. The synthesized USPIO-PAA/PEG had a good relaxation rate of 84.65 s^−1^ Mm^−1^, indicating that they could be efficiently uptake and traced by MRI. Furthermore, the primary human adipose-derived stem cells (HADSCs) were characterized, labeled with USPIO-PAA/PEG and transplanted into the striatum of 6-hydroxydopamine (6-OHDA)-induced PD rat models. The labeled cells could be traced by MRI for up to 3 weeks after the transplantation surgery; moreover, transplantation with the labeled HADSCs significantly attenuated apomorphine-induced rotations in PD models and increased the number of the dopaminergic neurons in the *substania nigra*. Overall, the development of USPIO-PAA/PEG NPs provides a promising tool for in vivo tracing technique of cell therapy and identifies a novel strategy to track stem cells with therapeutic potential in PD.

## Introduction

Parkinson’s disease is a neurodegenerative disease which is characterized by the progressive loss of dopaminergic neurons in the midbrain. The relatively focal impairment makes it a good candidate for cell-based therapies. Several cell types, ranging from fetal midbrain tissue [1] to induced neurons derived from embryonic stem cells (ESC) or induced pluripotent stem cells (iPSC) [2], have been implicated in the treatment of PD; however, ethical concerns and the potential risk of tumorigenicity could not be avoided during the application [3]. Mesenchymal stem cells (MSC) are multipotent stem cell population that has been reported in the last decade as a promising therapeutic tool for the neurodegenerative disease, including PD [4, 5]. Compared to other cell types, MSC show good proliferation, widespread availability throughout the human body and immunorepressant ability. Some studies have shown that intracranial transplantation of MSCs promote neuroprotection, neuronal differentiation and immunomodulation [6–8]. In these studies, tracking the viability, migration and integration of transplanted cells in vivo is essential for a basic understanding of stem cell function and evaluation of clinical effectiveness.

To better analyze the therapeutic effects of the transplanted cells, various tracers have been developed. The safety and efficacy of tracers are the most critical factors influencing the application. Compared to the labeling with fluorescent proteins [9], tracking using superparamagnetic iron oxide (SPIO) particles would not bring any genetic modification to the transplanted cells [10–12]. Several studies have used SPIO as an effective contrast agent to visualize and track the transplanted cells [6, 13, 14]. However, some of the commercial available SPIO have been reported to affect the normal functions of MSC [15–17]; therefore, extensive efforts have been devoted to develop novel SPIOs. In the present study, we synthesized an ultrasmall SPIO nanoparticles (USPIO) coating with polyacrylic acid (PAA) and a subsequent methoxypolyethylene glycol amine (PEG) layer. The novel USPIO-PAA/PEG shows good cellular internalization and long-term MRI tracking capacity in vitro and in vivo. Moreover, the USPIO-PAA/PEG-labeled MSCs derived from human adipose tissue (HADSCs) maintained the biological features, improving the behavioral impairments and increasing TH immunoreactivity in the substania nigra of PD animal models.

## Materials and Method

### Materials

Iron acetylacetonate, TREG (triethylene glycol), diethylene glycol and PEG were purchased from Aladdin Industrial Corporation (shanghai, China). Methylthiazolyldiphenyl-tetrazolium bromide (MTT) was purchased from Sigma-Aldrich Corporation (Shanghai, China). Dulbecco's modified eagle medium (DMEM), trypsin–EDTA (0.25%) and fetal bovine serum (FBS) were purchased from Thermo Fisher Scientific. 1-(3-Dimethylaminopropyl)-3-ethylcarbodiimide hydrochloride and N-Hydroxy succinimide were purchased from Sigma-Aldrich (China). N, N-Dimethylformamide, ethyl alcohol absolute and ethyl acetate were purchased from Sinopharm Chemical Reagent Corporation. Ultrapure water was produced by Millipore pure and ultrapure water purification systems. Antihuman CD90-FITC, antihuman CD45-PE, antihuman CD34-FITC and antihuman CD105-PE were purchased from Biolegend.

### Synthesis of USPIO

The USPIO was synthesized by polyol method as previous research [18–21]. Steps of experiment are as follows: iron acetylacetonate 1 mmol and TREG 25 mL were mixed in a three-neck flask, which connected to argon, spherical condensing tube and thermometer. After incubated with argon flow for 15 min, the mixture was heated to 120℃ (heating rate is 3 °C/min) and maintained for 1 h, and then the mixture was heated further to 250 °C at the same heating rate and maintained for 30 min. The reaction mixture was natural cooled to room temperature, diluted with 2 mL absolute ethyl alcohol, followed by precipitation with ethyl acetate. The sediment was collected by centrifugation at 8000 rpm for 20 min and then resuspended in absolute ethyl alcohol. The USPIO was stored in absolute ethyl alcohol at 4 °C for further use.

### Preparation of PAA/PEG Coated USPIO

The USPIO-PAA was acquired according to a previous report [22]. In detail, polyacrylic acid (PAA) 1.5 g was dissolved into 24 mL diethylene glycol. After a 5-min argon flow, the mixture was heated to 110 °C and maintained until the solution pellucid. The ethanol dispersing solution containing 28 mg of USPIO prepared in the previous step was added to the above clarified solution after ultrasonic dispersing. The solution was finally heated to 210 °C at a rate of 3 °C/min. The reaction was stopped after 2 h of reflux and naturally cooled to room temperature. After cooling, ethyl acetate was added to the reaction solution to precipitate the USPIO NPs and then centrifuged at 8000 rpm for 20 min to remove the supernatant. The precipitate was then dispersed in ethanol, followed by precipitation with ethyl acetate. After the washing procedures for three times, the USPIO-PAA was dispersed in ultrapure water for further use. In order to improve biocompatibility, we further linked PEG to the surface of nanoparticles by EDC and NHS-mediated amidation [23]. 0.45 g of PEG was dissolved in 5 ml ultrapure water and added to the 15 ml USPIO-PAA dispersion (contains 35 mg Fe) supplemented with 20 mg EDC and 12 mg NHS. The mixture was mechanical stirred at room temperature for 2 h, and then 10 ml DMF solvent was added, removing water at 40℃ in the rotary evaporation apparatus. Finally, 40 mg DEC and 24 mg NHS were added to the mixture, mixing at room temperature for 48 h. The reaction solution was transferred to a dialysis bag in ultrapure water for 72 h, during which ultrapure water was replaced every 12 h. The mixture was further transferred into an ultrafiltration tube to collect molecules with an Mr > 30 kD by centrifugation at 4000 rpm for 10 min. The final product was dissolved in ultrapure water and stored at 4 °C. In some cases, SPIO-PAA/PEG dissolving in water was collected by centrifugation with an ultrafiltration centrifuge tube (> 30 KD pore size, Millipore), and the pellets were dried in a vacuum oven for further experiment.

### Characterization of NPs

The particle size, size distribution and morphology of the samples were analyzed by TEM. The coating layer was measured by negative staining. After ultrasonic dispersion, the USPIO-PAA and USPIO-PAA/PEG in aqueous solution were added to the copper network of 300-mesh carbon supporting membrane, dried naturally and then put into a projection electron microscope (Tecnai G2F20 s-twin, FEI) for observation.

The infrared spectroscopy analysis is performed on Agilent Technologies Cary 600 Series FTIR Spectrometer, where the dried sample powder is added directly to the sample tank for detection.

The Fe content in USPIO-PAA/PEG dispersion was determined by flame atomic absorption spectrometry (FAAS), whose model is PerkinElmer Analyst 700. First, 1, 2, 3, 4 and 5 μg/mL iron standard solution was configured to draw the standard curve, and then 100 μL USPIO-PAA or USPIO-PAA/PEG dispersion solution was dissolved with nitric acid, and the content of iron was determined in a 50-mL volumetric flask.

Magnetic resonance imaging of the nanoparticles was conducted on clinical scanners with magnetic field of 3 T in the imaging department of the first affiliated hospital of Soochow University. The nanoparticles were dispersed in 1% agarose gel solution according to the corresponding concentration. After the solution solidified, the transverse relaxation time of the samples was measured by multi-echo sequence. Exporting the acquisition of MRI in DICOM format and then using the RadiAnt DICOM Viewer to open, read the grey value, by importing the gray values of different echo times into origin for fitting, the transverse relaxation time values of USPIO dispersions with different concentrations were obtained; finally, the transverse relaxation rate r2 of USPIO-PAA and USPIO-PAA/PEG samples was obtained by linear fitting with the transverse relaxation rate of 1/T2 and the Fe concentration of the samples. The magnetic hysteresis loops of USPIO-PAA and USPIO-PAA/PEG were acquired by using Quantum Design DynaCool-9 device in Suzhou University of science and technology according to the methods described previously [24].

### HADSCs Isolation and Identification

All studies were done in accordance with the ‘Ethical Guiding Principles on Human Embryonic Stem Cell Research’ (of the Ministry of Science and Technology and the Ministry of Health, People’s Republic of China, 2003) and Helsinki Declaration. Adipose samples were obtained with informed consent and ethical approval from the first affiliated hospital of Soochow University. The adipose tissue was washed and cut into small pieces after removing the blood vessels and connective tissue under an anatomical microscope. The blocks were digested with type I collagenase (0.3 pzu/mL) at 37 °C for 30 min, followed by centrifugation at 500*g* for 10 min. The cell pellet was suspended with in DMEM + 10% FBS and seeded at the density of 8 × 10^4^/cm^2^. After 48 h, the old medium containing floating cells was discarded and replaced with fresh medium.

HADSCs were characterized by flow cytometry for surface markers specifically labeling mesenchymal (CD90 and CD105) and hematopoietic (CD34 and CD45) stem cells. A total of 1 × 105 cells were harvested and incubated with either PE, FITC, APC/cy7 or PerCP-conjugated antibodies against CD34, CD45, CD90and CD105 mouse antihuman monoclonal antibodies and appropriate isotype controls. Stained cells were analyzed using a flow cytometer (LSRFortessa, BD, USA), and data were analyzed using FlowJo software. Four phenotypes of CD90+, CD105+, CD34− and CD45− were selected for the surface markers of HADSCs. Expression levels of cell surface markers were identified by flow cytometry (BD LSRFortessa).

The adipogenic and osteogenic differentiation of HADSCs were evaluated by Oil Red O and Alizarin Red Staining, respectively. HADSCs were cultured with either MesenCultTM Adipogenic Differentiation medium (Stem cell Tech., 05412) or OriCellTM Osteogenic Differentiation Kit (Cyagen, HUXMA-90021). For adipogenic differentiation of HADSCs, cells were assessed on day 14 by qualitative Oil Red O staining for lipid-filled mature adipocytes (VivaCell Biosciences, C37A00150). For osteogenic differentiation of HADSCs, cells were assessed on day 21 by Alizarin Red Staining for calcium nodule in mature osteocytes (VivaCell Biosciences, C37C00150). Images were acquired using an inverted Nexcope microscope.

### In Vitro Cellular Uptake of USPIO-PAA/PEG and Biocompatibility Evaluation

HADSCs were plated in a 6-well plate with a density of 1 × 10^6^ per well. Cells were incubated with the medium containing USPIO-PAA/PEG (Fe 10 μg/mL and 0.1 μg/mL) for 2 hs, and stained with Prussian blue (PB) staining for intracellular Fe identification.

The biocompatibility of USPIO-PAA/PEG was determined by MTT colorimetry and 5-ethynyl-2′-deoxyuridine (EdU) incorporation assay (EdU proliferation kit, Thermo). For MTT, HADSCs were plated in a 96-well plate at a density of 5 × 10^3^ per well and incubated with different concentrations (0, 10, 20, 40, 80, 160 Fe μg/mL) of USPIO-PAA/PEG 24 h, 48 h or 72 h. 20 μl MTT solution (5 mg/mL) was added to each well for 4 h, followed by 150 μL dimethyl sulfoxide to dissolve the crystals. Absorbance value of each hole was measured at OD 570 nm using enzyme-labeled instrument (BioTek Synergy HT). For EdU assay, HADSCs were incubated with the SPIO-PAA/PEG at a 160 Fe μg/mL for 72 h, followed by the incubation with 10 μM EdU for 24 h. Cells were collected, fixed with 4% paraformaldehyde and neutralized with a 2 mg/mL glycine. After permealization cells were incubated with the dye solution (Azide 647 complex), the EdU incorporation rates were evaluated by flow cytometry.

### 6-OHDA-Induced PD Model

Fifteen–twenty of male Wistar rats (SPF grade, weighing 220 ± 20 g) were used for in vivo tracing of USPIO-PAA/PEG-labeled HADSCs on PD animals. All procedures were performed according to the Regulations in China (Regulations for the administration of affairs concerning experimental animals, 2017) and approved by the Institutional Animal Care and Use committee at Chinese Academy of Sciences. Throughout, animals were housed under controlled illumination (12/12-h light/dark cycle, “on” at 7 a.m.) with ad libitum access to food and water. PD model was prepared by 2-point injection of 6-hydroxydopamine (6-OHDA, Sigma, St. Louis, MO, USA) into the unilateral striatum of rats. As described previously [25, 26], rats were stereotaxically injected with 3 μL of 6-OHDA solution (5 μg/μL) at 2 coordinates (AP: 1.2 mm, ML: 2.2 mm, DV: − 4.0 to 6.0 mm; and AP: − 1.0 mm, ML: 4.4 mm, DV: − 4.5 to 6.5 mm), respectively. Apomorphine-induced rotation (0.5 mg/kg, subcutaneously) tests were used to test the validity of PD models. The rats were injected with apomorphine (0.5 mg/kg) subcutaneously at 1, 2 and 3 weeks following 6-OHDA treatment, and the rotation scores were evaluated for 30 min in an open field. The PD model has more than 7 rotations per min induced by apomorphine and was considered succeed.

### Cell Transplantation and In Vivo MRI Imaging

HADCs were incubated with USPIO-PAA/PEG (iron concentration was 10 μg/mL) for 2 h when they reached 80% confluence. 3 weeks after PD model preparation, 3 × 10^6^ of USPIO-PAA/PEG-labeled HADCs or saline were injected into the left striatum of PD rat models at the following coordinate (AP: 1.2 mm, ML: 2.2 mm, DV: − 4.0 to 6.0 mm). Animals received transplantation were administrated with buprenorphine (0.5 mg/kg) immediately after surgery and for 2 days thereafter. These animals were subjected to the apomorphine-induced rotation tests at 1, 2 or 3 weeks after the transplantation surgery.

For in vivo MRI, animals were imaged using an in vivo imaging system (IVIS) small animal imaging system (PerkinElmer, Waltham, MA, USA) at the 3th, 9th, 15th and 21th days following the saline or HADSCs injection.

### Histology Analysis

The brains of the remaining rat models were harvested and sectioned to a thickness of 30 mm for analysis 3 weeks post-stem cell transplantation (*n* = 5 per group). Sections were incubated with anti-tyrosine hydroxylase (TH, abcam, England) and visualized with Alexa-594-conjugated donkey anti rabbit antibody (Abcam). DAPI was used for counterstaining with nuclei. Images were captured using Leica TCS SP5 confocal microscope.

### Statistical Analysis

Numerical data were expressed as the mean ± SD. Data were subjected to 2-tailed Student t tests or one-way ANOVA using GraphPad Prism 7.0 (GraphPad Software Inc., San Diego, CA, USA) to evaluate the difference. * indicates *p* < 0.05 and NS indicates no significant difference.

## Results

### The Synthesis and Characterization of Nanoparticles

**Fig. 1:**
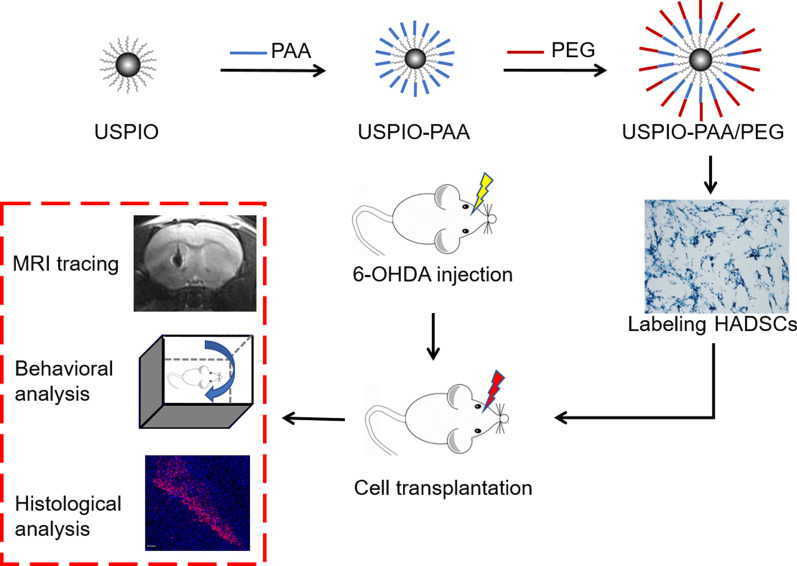
Schematic illustration of the experimental design

**Fig. 2:**
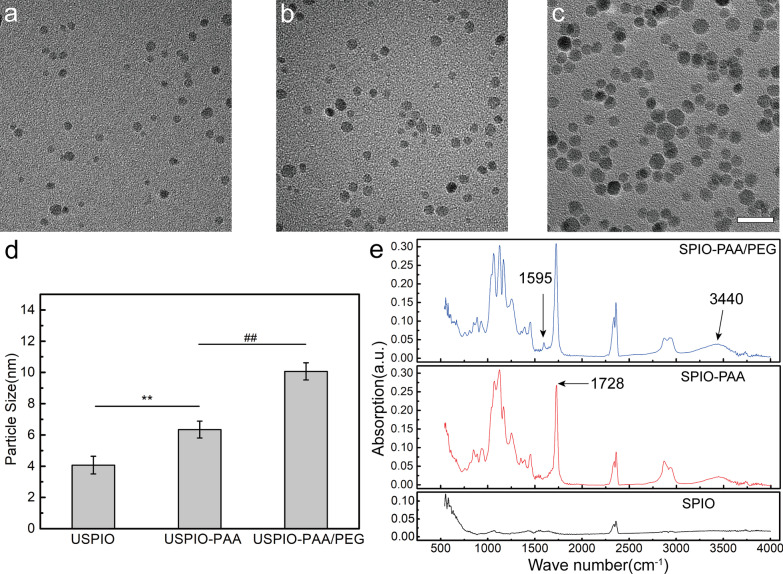
Characterization of nanoparticles. **a–c** Representative TEM images of USPIO NPs (**a**), USPIO-PAA NPs (**b**) and USPIO-PAA/PEG NPs (**c**). **d** Statistical analysis indicated a significant difference among the particle size of USPIO, USPIO-PAA and USPIO-PAA/PEG (*n* = 20 per group). **e** Fourier-transform infrared of nanoparticles of USPIO, USPIO-PAA and USPIO-PAA/PEG NPs. The arrows indicate the specific absorption peak at 1728 cm^−1^ due to the PAA modification, 1595 cm^−1^ and 3440 cm^−1^ due to the PEG modification. Numerical data are presented as mean ± SD, **p < 0.01 versus the USPIO NPs, ##*p* < 0.01 versus the USPIO-PAA/PEG NPs. The bar represents 20 nm

The experimental design is shown in Fig. 1. The hydrophobic USPIO NPs were prepared by using the thermal decomposition method. As shown in Fig. 2a–c, the diameter of the original USPIO NPs was 4.07 ± 0.57 nm; by coating with PAA, the diameter of the USPIO-PAA NPs increased to 6.34 ± 0.54 nm; a further modification of the NPs with PEG makes the diameter of USPIO-PAA/PEG NPs reach 10.07 ± 0.55 nm. All three nanoparticles were spherical in shape and evenly dispersed. Statistical analysis showed that there was significant difference (*F* = 10, **p < 0.01, ##*p* < 0.01) among the diameters of the USPIO, USPIO-PAA and USPIO-PAA/PEG NPs, indicating a successful modification with PAA and PEG (Fig. 2d).

By using the infrared absorption method, we found that USPIO-PAA has an obvious carbonyl absorption peak at 1728 cm^−1^ due to the carbonyl stretching vibration peak in the PAA molecule; USPIO-PAA/PEG has a carbon–nitrogen bond in-plane bending vibration peak at 1595 cm^−1^ and a stretching vibration peak of hydroxyl group at 3440 cm^−1^ (Fig. 2e). All of these data further confirmed that PAA or PEG molecules have been successfully modified to the surface of NPs.

### USPIO-PAA/PEG Showed Good In Vitro Magnetic Response and Biocompatibility with HADSC

**Fig. 3:**
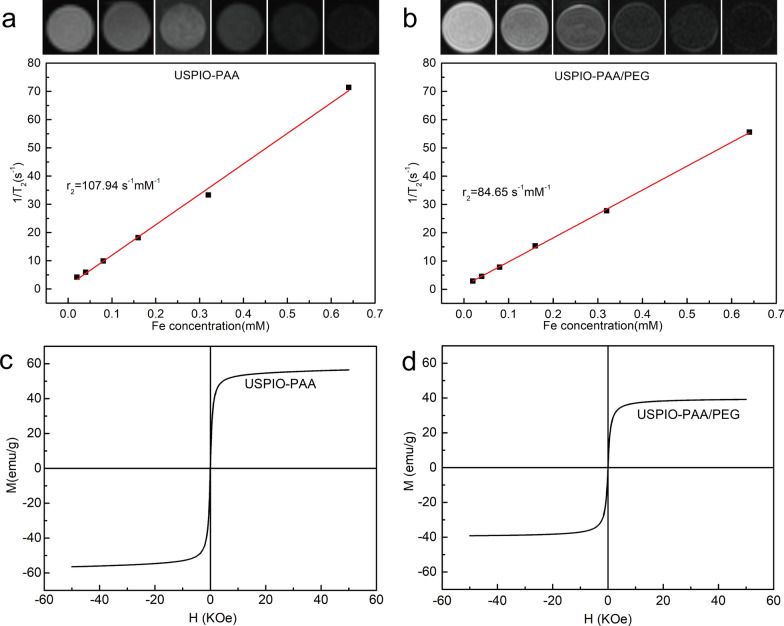
In vitro magnetic response of the synthesized USPIO-PAA and USPIO-PAA/PEG NPs. **a**, **b** MRI and transverse relaxation rate of USPIO-PAA NPs (**a**) and USPIO-PAA/PEG NPs (**b**). *r*_2_ represents relaxation rate. The relaxation rates of the USPIO-PAA and USPIO-PAA/PEG NPs were 107.94 s^−1^ mM^−1^ and 84.65 s^−1^ mM^−1^. **c**, **d** magnetic hysteresis loops of USPIO-PAA NPs (**c**) and USPIO-PAA/PEG NPs (**d**). The magnetic hysteresis was measured at 295 k (21.85 °C) with the maximal magnetic field intensity 50,000 Oe (5 Telsa). The saturation magnetization values were close to 57 emu/g for SPIO-PAA and 40 emu/g for SPIO-PAA/PEG. Magnetization hysteresis in both curves was close to zero

The in vitro imaging ability of USPIO-PAA and USPIO-PAA/PEG was evaluated by the grayscale values of MRI images at different concentrations (equivalent to Fe concentrations). After fitting, the transverse relaxation time values of the dispersions at different concentrations were obtained. By setting the transverse relaxation rate of 1/T2 as the vertical coordinate and the Fe concentration as the horizontal coordinate, the transverse relaxation rate of USPIO-PAA was calculated as 107.94 s^−1^ mm^−1^ (Fig. 3a), and 84.65 s^−1^ mm^−1^ for USPIO-PAA/PEG (Fig. 3b). Both particles showed good MRI imaging properties. The magnetic hysteresis loops of SPIO-PAA and the SPIO-PAA/PEG showed that the saturation magnetization value of SPIO-PAA is 57 emu/g above 10,000 Oe (Fig. 3c), and the one of SPIO-PAA/PEG is 40 emu/g above 10,000 Oe (Fig. 3d). The coercive force and remanence were close to zero (Fig. 3c, d), further indicating that both SPIO-PAA and SPIO-PAA/PEG were superparamagnetism.

**Fig. 4:**
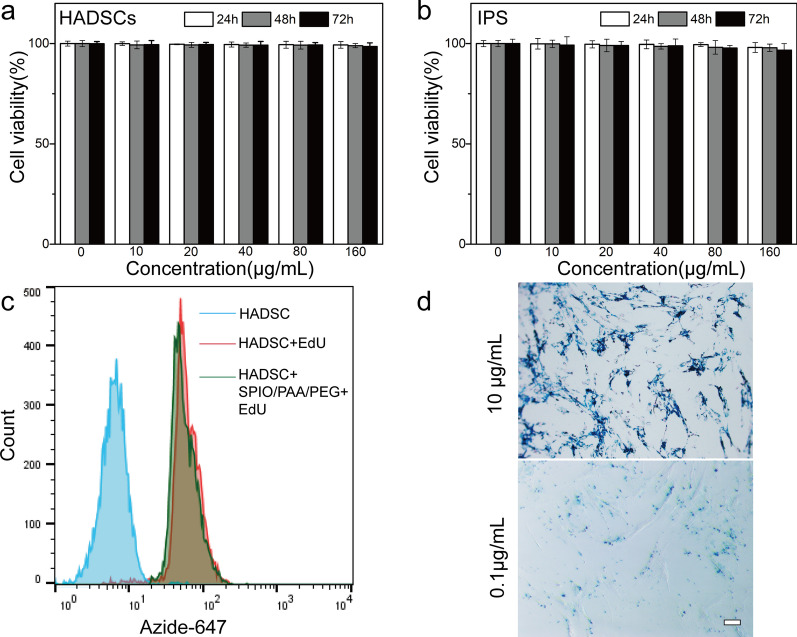
The synthesized USPIO-PAA/PEG NPs show good biocompatibility and labeling capability. **a**, **b** HADSCs or iPSCs were incubated with USPIO-PAA/PEG NPs at varying concentrations (equivalent to Fe concentrations at 0, 10, 20, 40, 80 and 160 μg Fe/mL) for 24, 48, and 72 h, and the cell viability was determined by MTT method and shown in **a**, **b**, respectively. There is no significant difference among the viabilities at different NPs concentration (*n* = 5) or incubation time (*n* = 5). **c** Flow cytometry analysis showed that the EdU incorporation rates were similar in the HADSCs incubated in the presence or absence of USPIO-PAA/PEG (160 μg Fe/mL for 72 h). HADSCs did not treated with EdU were served as negative control. **d** USPIO-PAA/PEG at 10 μg Fe/mL and incubation time for 2 h resulted in almost 100% labeling efficiency of HADSCs, as evaluated by Prussian blue staining. Note that USPIO-PAA/PEG at 0.1 μg Fe/mL (2 h incubation time) produced light but uniform PB stain in the HADSCs. Numerical data are presented as mean ± SD. The bar represents 20 μm

By incubating the USPIO-PAA/PEG NPs with HADSCs for different time, we found that USPIO-PAA/PEG NPs has no significant effects to the survival of cells (*p* > 0.05). The cell viability of HADSCs remains above 96% when the equivalent Fe concentration ranged from 0 to 160 μg/mL. Moreover, the increase of the incubation time (from 24 to 72 h) does not induce significant cell loss at each Fe concentrations (Fig. 4a). A similar observation was obtained when USPIO-PAA/PEG NPs were incubated with iPSCs at different concentrations (Fig. 4b). Eventhough HADSCs were treated with the 160 μg Fe/mL USPIO-PAA/PEG for 72 h, the proliferation ability, as indicated by the EdU incorporation rates, has not impaired by the presence of USPIO-PAA/PEG (Fig. 4c). All of these data indicated the biosafety of the synthesized USPIO-PAA/PEG NPs.

To identify whether cells could be effectively labeled by USPIO-PAA/PEG NPs, we have incubated the HADSCs with USPIO-PAA/PEG NPs at equivalent Fe 10 μg/mL or 0.1 μg/mL for 2 h. As shown in Fig. 4d, a large number of NPs stained into blue were observed in almost 100% of HADSCs in the presence of USPIO-PAA/PEG (Fe 10 μg/mL). Eventhough the dose of USPIO-PAA/PEG reduced to 0.1 μg Fe /ml, we still observed rather uniform and dispersed PB staining in almost all of the HADSC. As compared to some of the other reported SPIOs [14, 27, 28], USPIO-PAA/PEG exhibited high cell uptake efficiency.

### Long-Term Tracking of USPIO-PAA/PEG NPs-Labeled HADSCs in PD Rat Models

**Fig. 5:**
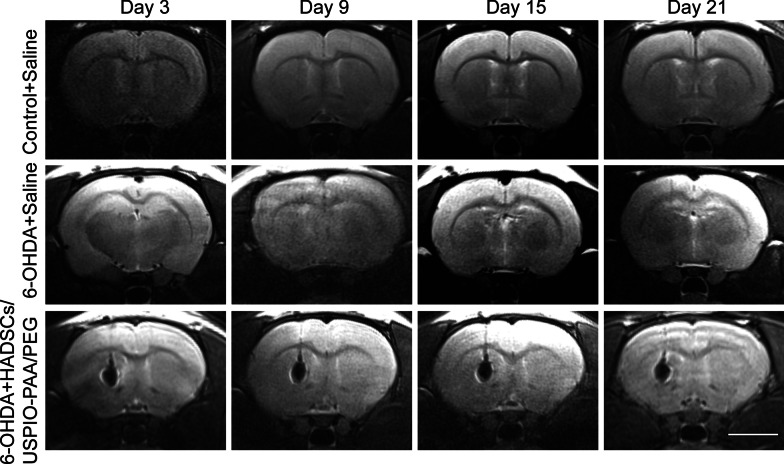
Representative MRI images of the brains at 3 days, 9 days, 15 days and 21 days followed by transplantation with USPIO-PAA/PEG labeling HADSCs or saline. The MRI images of the corresponding controls, i.e. normal rats or PD rat model injected with saline, were shown in the 1st and 2nd rows, respectively. Note that compared to the negative signals in the control, the rat brains transplanted with USPIO-PAA/PEG-labeled HADSCs showed a constant and clear MRI signals up to 21 days. The bar represents 5 mm

HADSCs labeled with USPIO-PAA/PEG NPs were transplanted into the left striatum of PD rat model by stereotactic injection. As shown in Fig. 5, we observed a clear MRI signal in the left striatum 3 days after the HADSC transplantation, indicating a proper in vivo tracing of the transplanted cells. A dynamic observation on D3, D9, D15 and D21 after transplantation showed that the transplanted cells with NP labeling could be clearly traced up to 3 weeks, and the MRI signals did not show obvious reduction during the process. These results indicate that the synthesized nanoparticles have good potential for the in vivo tracing of the transplanted cells.

### USPIO-PAA/PEG NPs-Labeled HADSCs Exhibited Therapeutic Effects in PD Model

**Fig. 6:**
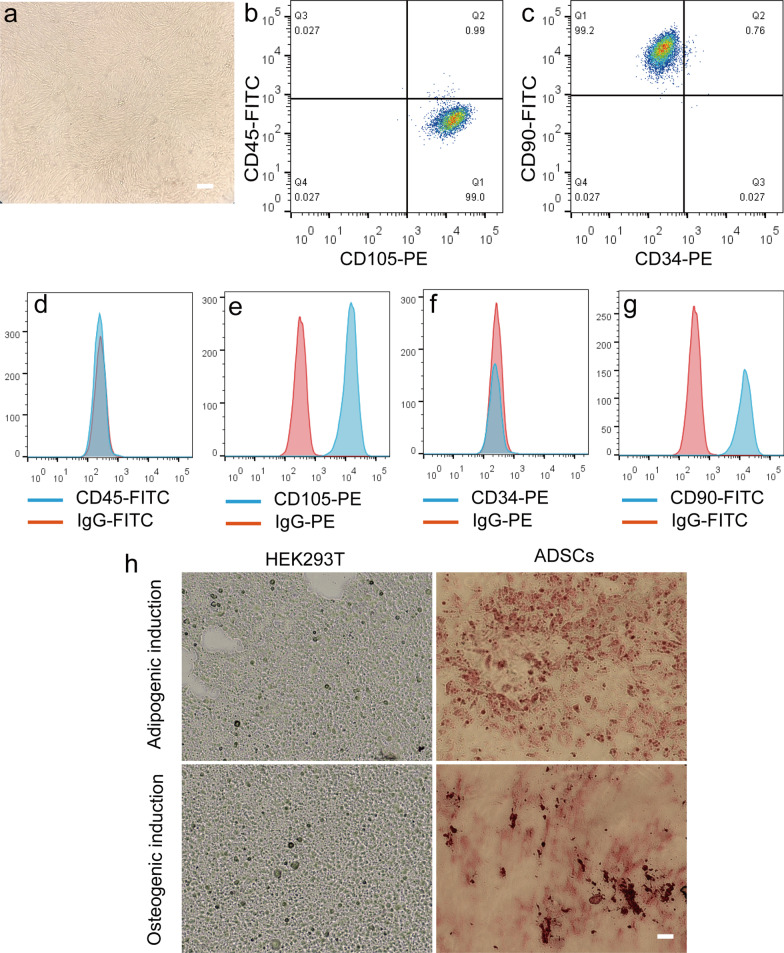
HADSCs characterization. **a** A representative image under the phrase contrast microscope showing that HADSCs has a typical spindle-like phenotype. **b–g** HADSCs were incubated with antibodies conjugating with different dyes against CD45, CD105, CD34 and CD90 markers and subjected to flow cytometric analysis. The scatter diagrams are shown in **b, c**, and the histograms are shown in **d–g**. Note that most of the cells were expressed CD90 and CD105, but not CD34 and CD45. **h** Adipogenic differentiation and osteogenic differentiation of HADSCs versus HEK293 cells are evaluated by Oil Red O or Alizarin Red Staining, respectively. The bar represents 20 μm

To evaluate whether NPs-labeled HADSCs exhibited therapeutic effects, we first performed a phenotypic characterization of the isolated HADSCs. As shown in Fig. 6a, the cultured HADSCs were spindle-shaped cells. The flow cytometry analysis showed that the HADSCs were highly expressed markers for mesenchymal cells, such as CD90 (> 99%) and CD105 (> 99%), whereas few of them expressed hematopoietic markers such as CD34 and CD45 (Fig. 6b–g). The HADSCs could differentiate towards either adipogenic or osteogenic lineages under certain conditions, and the differentiation capacity was shown by Oil Red O or Alizarin Red Staining, respectively (Fig. 5h). Note the lipid droplets or calcified nodules in the differentiated HADSCs, while no such structure in the control HEK293 cells (Fig. 6h). All of these indicate that HADSCs fulfill the criteria of mesenchymal cells.

**Fig. 7:**
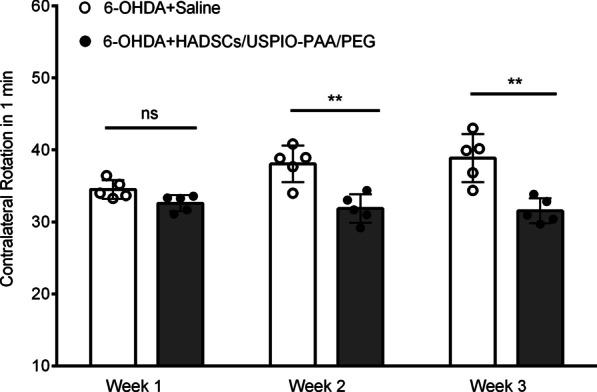
PD rat models were administered with either saline or USPIO-PAA/PEG-labeled HADSCs in the ipsilateral striatum impaired with 6-OHDA, and the rotation numbers per min were scored and compared. There is significant reduction of the rotation numbers in the 2nd, 3rd weeks after the PD rats receiving HADSCs, as compared to the corresponding PD rats receiving saline. *ns* indicates insignificant change, and ** indicates *p* < 0.01. *n* = 5 for each group

The apomorphine-induced rotations were 17.1 ± 1.79, 28.7 ± 1.77 and 31.86 ± 1.72 per min in the 1st, 2nd and 3rd weeks after 6-OHDA injection, confirming the successful establishment of the rat PD model. As shown in Fig. 7, the rotation numbers of PD rat models reduced to 32.59 ± 1.12, 31.85 ± 1.98 and 31.54 ± 1.73 in the 1st, 2nd and 3rd weeks followed by NPs-labeled HADSCs transplantation in the striatum of lesion side. Statistical analysis revealed a significant difference between the saline- and the HADSC-administered groups from the 2nd week after transplantation surgery (*p* < 0.01 at 2nd week, *p* < 0.01 at 3rd week; *n* = 5 in each group), indicating the NPs-labeled HADSCs improves the behavioral impairments in the PD models.

**Fig. 8:**
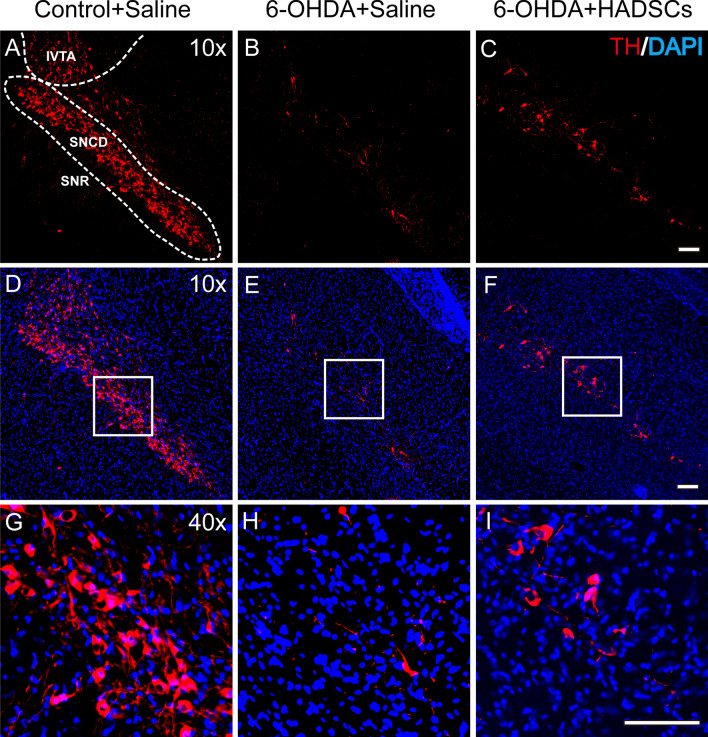
Immunostaining against TH in the substantia nigra of the rats receiving saline (**a**, **d**, **g**), PD rats receiving saline (**b**, **e**, **h**) or PD rats receiving USPIO-PAA/PEG NPs-labeled HADSCs (**c**, **f**, **i**). The area in the dashed frame indicates the compact area of substantia nigra (SNc) and the ventral tegmental area (VTA). Images in the boxes of **d**, **e**, **f** are enlarged and shown in **g**, **h**, **i**, respectively. There are less TH-expressing cells in the SNc and VTA regions in the rats receiving 6-OHDA injection, as compared to the controls receiving saline. Following by HADSC transplantation, more of TH-expressing cells in the SNc of PD rats could be observed. The bar represents 100 μm

PD is characterized by the loss of neurons expressing tyrosine hydroxylase (TH) in the substantia nigra. By using immunostaining against TH, we found that 6-OHDA reduces the TH-expressing neurons in the substantia nigra, and the transplantation with HADSCs in the striatum of PD rat models could alleviate such reduction (Fig. 8). Combined with the observation that cell transplantation alleviates the behavioral impairments of PD rats, we concluded that USPIO-PAA/PEG NPs-labeled HADSCs exert therapeutic effects in PD rat models.

## Discussion

Cell therapy is a promising strategy for the treatment of neurodegenerative diseases and has been in the front edge of preclinical research over the last 20 years [29, 30]. Tracing the transplanted cells is an indispensable part for the clarification of underlying mechanisms as well as the evaluation of clinical effects; however, it is challenging and to some extent, hampered the application of cell therapy [31, 32]. Computed tomography (CT), near-infrared fluorescence imaging (NIFI) and MRI are the most commonly used methods for the in vivo tracking of transplanted cells [31, 32]. Compared to the rather high radiation of CT and low sensitivity of NIFI, MRI shows good imaging of deep tissue, high contrast and low ionizing radiation, making it a good candidate for the in vivo tracing [33–36]. The development of proper tracers for MRI therefore becomes an indispensable part of promoting the application of cell therapy.

SPIO is a simple and reliable labeling strategy for MRI visualization. To achieve long-term in vivo tracing of the transplanted cells, the SPIO particles is required to have a uniform and ultrasmall size, since large particles may lead to uneven distribution of the tracers and interfere with the normal blood circulation [33]. Moreover, the internalization efficiency and biocompatibility of nanoparticles are another important index to evaluate the tracers. Nanoparticles usually enter cells through liquid phase endocytosis [37], receptor-mediated endocytosis [38] and phagocytosis [39]. To achieve a better cellular uptake, the SPIO particles are usually modified with intermediate ligands [18, 40, 41].

Besides cell tracking, SPIO has also been applied for drug delivery [42]. The tissue distribution and pharmacokinetic clearance are important index for both applications. Compared to the NPs-PAA, NPs-PAA/PEG showed longer retention time in blood and slower urinary clearance [43]. NPs densely coated with PEG provide much more uniform distribution over mucosal epithelial surfaces, including the gastrointestinal tract [44], respiratory system [45, 46] and brain tumor [42], etc. Moreover, PEG coating reduces the attachment of proteins and formation of corona, which in turn might promote uniform distribution of the NPs at the cost of reduced uptake [47]. As a potential good diagnostic and therapeutic tool, the labeling capacity of SPIO-PAA/PEG to MSCs and its further application in brain disorders such as PD have not been well studied, and the present study was designed to answer this question. In the present study, we synthesized a novel ultrasmall USPIO-PAA/PEG nanoparticles, modifying the SPIO cores with PAA and PEG ligands, respectively. The USPIO-PAA/PEG NPs have uniform ultrasmall diameter (10.07 ± 0.55 nm), good dispersion in aqueous solution, biocompatibility with various cell types as well as good magnetic response effect in vitro and in vivo. Importantly, the signals of labeled HADSCs could be clearly detected under MRI after brain transplantation for up to three weeks, indicating the good potential for clinical application.

PD is characterized by a progressively loss of dopaminergic neurons in the substania nigra, and by the deficiency of dopaminergic levels in the dopaminergic networks [1]; the most affected one is the nigrostriatal pathway including the striatum. In the present study, we transplanted USPIO-PAA/PEG-labeled HADSCs into the striatum of PD rat models, and found that such transplantation improved the behavioral impairments; moreover, it attenuated the loss of dopaminergic neurons in the substania nigra to some extent. Similarly, Ardeshir et al. reported that transplanting the MSCs derived from rat adipose tissues attenuated apomorphine-induced rotations in PD models [48]. Different from transplanting fetal midbrain tissues [49] or dopaminergic neural progenitors [50], HADSCs exert its therapeutic effects mainly through the paracrine effects [51, 52], rather than substituting for the impaired tissues. Studies have shown that MSCs act as promoters of immunomodulation, neuroprotection and neuronal differentiation, and these effects are essentially mediated by the secretome released by MSCs [6, 7, 51]. Our result is consistent with these observations; moreover, it indicates that the novel USPIO-PAA/PEG tracers did not interfere with the neuroprotection effects of HADSCs.

## Conclusions

We have developed a novel USPIO-PAA/PEG tracers showing high cell uptake efficiency, excellent biocompatibility and long-term MRI tracing capacities. The HADSCs labeled with USPIO-PAA/PEG could be traced with MRI for 3 weeks after cell transplantation. Moreover, the labeled HADSCs significantly attenuated the behavioral impairments of PD models, and increase the number of dopaminergic neurons in the substantia nigra. The development of USPIO-PAA/PEG tracer may provide a promising tool in stem cell research and application.

## Data Availability

All data supporting the conclusions of this article are included within the article.

## Abbreviations

SPIO: Small superparamagnetic iron oxide
USPIO: Ultrasmall superparamagnetic iron oxide
PAA: Polyacrylic acid
PEG: Methoxypolyethylene glycol amine
HADSCs: Human adipose-derived stem cells
USPIO-PAA/PEG: Ultrasmall superparamagnetic iron oxide nanoparticles coating with the polyacrylic acid and methoxypolyethylene glycol amine
6-OHDA: 6-Hydroxydopamine
CT: Computed tomography
NIFI: Near infrared fluorescence imaging
MRI: Magnetic resonance imaging
TH: Tyrosine hydroxylase
TEM: Transmission electron microscope
